# Integrating Well-Being in Living and Learning Through Occupational Therapy Fieldwork on a College Campus: The WILL Thrive Program

**DOI:** 10.3390/bs16040601

**Published:** 2026-04-17

**Authors:** Sarah E. Brockway, Carrie Will, Molly Mendolia, Karen Keptner

**Affiliations:** 1 Department of Occupational Therapy, Russell Sage College, Troy, NY 12180, USA; 2 Office of Student Disability Services, Colgate University, Hamilton, NY 13346, USA; mmendolia@colgate.edu; 3 Department of Occupational Therapy, Southern California University of Health Sciences, Whittier, CA 90604, USA

**Keywords:** occupational therapy, higher education, student mental health, student well-being, campus well-being, neurodiversity, health promoting campus, program feasibility, accessibility services, fieldwork education

## Abstract

Postsecondary institutions are seeing an increased prevalence of student mental health concerns and disabilities, highlighting the need for campus-based approaches that support student well-being. While college campuses provide many services to support students, occupational therapists are largely absent from these support systems, despite growing interest in this emerging field of practice. This program description and implementation case study examines preliminary indicators of feasibility for the WILL Thrive program, which delivered occupational therapy (OT) services on a college campus through a Level II fieldwork placement. Feasibility was examined across domains of acceptability, demand and implementation using an integrated approach combining a needs assessment, service development and process evaluation. Data sources included environmental observations, surveys, stakeholder interviews and process evaluation measures, including service delivery tracking, referral patterns, and resource utilization. Referrals and service utilization in this case were most frequently observed among students reporting neurodevelopmental and mental health-related functional challenges, providing preliminary indicators of potential service users, though a small, heterogeneous sample size limits generalizability. Referral patterns and engagement from the wellness center and accessibility staff highlight preliminary strengths of the program, including early indicators of acceptability and demand. In contrast, implementation barriers were also identified, including limited campus-wide understanding of the OT scope and role and constraints in on-campus OT supervision. Findings offer early, exploratory signals of feasibility for integrating OT services through an OT fieldwork II model and suggest that OT may complement existing campus supports by addressing participation-focused, functionally orientated needs. Results should be viewed as preliminary and inform future implementation studies that include systematic outcome measures, comparative analysis with existing services, and broader assessment across diverse higher education contexts.

## 1. Introduction

Postsecondary institutions are recognizing the need to holistically support student well-being and successful participation in college life (Bannigan et al., 2025; Worsley et al., 2022). At the same time, there has been an increasing number of students on campuses diagnosed with attention-deficit/hyperactivity disorder (ADHD) (Nash-Luckenbach & Friedman, 2024), autism spectrum disorder (ASD) (Zeedyk et al., 2019), and mental health conditions like anxiety (Auerbach et al., 2016; Lipson et al., 2019). Regardless of disability status, the transition to higher education presents significant occupational challenges that impact a student’s ability to balance academic demands with daily living, social participation, and self-care (K. M. Keptner & Rogers, 2019), ultimately impacting their well-being.

Occupational therapy practitioners (OTPs) possess a unique perspective on student well-being, supporting student well-being by using a whole person–environment approach and addressing barriers to student academic success through daily living needs (Nagata et al., 2022; K. Keptner et al., 2026a). *Integrating Well-Being in Living and Learning to Thrive* (WILL Thrive) is an occupational therapy-led pilot program delivered during a 12-week occupational therapy Level II Fieldwork (OT FWII) placement. The intent of the pilot was to identify areas on campus where occupational therapy (OT) could enhance established campus services across living and learning contexts. The intervention was designed in response to a needs assessment, through which data were collected to guide service development and process evaluation. The purpose of this program description and implementation case study is to describe the development process and examine the demand and acceptability of OT services on campus, how OT services could be implemented on campus, and the feasibility of providing OT services through an occupational therapy Level II fieldwork experience (OT FWII).

### 1.1. Occupational Therapy in Higher Education: Impacts on Student Success

College students face increasing demands on their occupational performance across academic, social, and daily living domains. Challenges with time management, social participation, emotional regulation, and self-advocacy affect student success regardless of diagnostic status. While students with identified disabilities such as ADHD, autism spectrum disorder, and anxiety may experience these challenges more acutely, research indicates that many students struggle with similar functional barriers without formal diagnosis or accommodation (Anderson et al., 2017; Henning et al., 2022; Palomares Gómez et al., 2023).

Occupational therapists work with people of all ages and with a wide variety of needs, ranging from physical disabilities to neurological conditions to mental health issues that are primary or secondary to their disability or diagnosis (American Occupational Therapy Association, 2021). OTPs working with college students can help improve a student’s experience of well-being, living and learning (Hunt et al., 2025) and help improve self-determination, defined as having agency to preserve or improve upon one’s quality of life (Hojati Abed et al., 2021). Eichler and Keptner (2023) report that OTPs working in higher education use a wide range of treatment interventions, including but not limited to life skills training, coping skills and anxiety management. OTPs take into consideration the environmental or social factors that may aid or impede therapy goals, as well as the client’s performance patterns, performance skills and client factors (American Occupational Therapy Association, 2020). Some notable examples of OT services used on campus include: Trinity College Dublin (TCD) and California Institute of Technology (CalTech). TCD, for example, employs OTPs within its disabilities office to find reasonable academic accommodations for students while using an OT lens and has been doing so for more than 20 years (Lewis, 2025). California Institute of Technology (Caltech) hires OTPs for its wellness department to offer OT as a student service (Ho, 2022). While general student support staff may be able to offer one-size-fits-all strategies for time management to students, OTPs can offer a multifaceted and evidence-based approach to student success and well-being (K. Keptner et al., 2026a).

### 1.2. OT Fieldwork on a College Campus

An Occupational Therapy Level Two fieldwork (OT FWII) placement is a requirement for OT graduate students, which allows students to apply their knowledge and skills learned in the classroom by providing OT services to clients under the supervision of a licensed OTP (Gupta & Amini, 2018). OT FWII students are often placed in hospitals, outpatient clinics, school districts, and home health. While there is an emerging trend to have OTPs work with college students to enhance their occupational well-being during their transition to college, there are currently no guidelines or defined roles for OTPs in higher education (Eichler & Keptner, 2023). Placing an OT FWII student on a college campus to work with college students creates a potential to normalize and support the addition of OTPs in service roles in tertiary learning environments. Finding ways to create roles for OTPs on campus will also help reduce the gaps in support for students with histories of ASD, ADHD or anxiety, who may encounter changes in support structures during the transition to postsecondary education, particularly after receiving structured services throughout their grade school years. Additionally, peer support has been shown to positively influence students’ perception of their academic competence (Worley et al., 2023); in this context, an OT FWII student may provide a uniquely relatable, peer-informed perspective drawing on their recent experience navigating academic and college-related challenges.

### 1.3. Theoretical Framework in Development of WILL Thrive

Two frameworks were utilized in the development of the WILL Thrive program: the Okanagan Charter and the PEOP Model. Implemented within existing campus supports through accessibility and wellness services, the program integrated well-being practices using an occupational lens and aligned with the Okanagan Charter’s fourth key principle of action, which emphasizes developing “trans-disciplinary collaborations and cross-sector partnerships” (Okanagan Charter, 2015, p. 9). Accordingly, collaboration among the college’s occupational therapy department, student wellness center, and accessibility services supported the Charter’s call for the “development of whole campus action for health and the creation of knowledge and action for health promotion” (Okanagan Charter, 2015, p. 9).

The Person–Environment–Occupation–Performance (PEOP) model is an occupational therapy-based theoretical framework that acknowledges the interconnectivity between a person, their occupations and their environment and how that relationship affects occupational performance (Bass et al., 2024). The PEOP model was selected for this program as it aligns with the college’s commitment to being a Health Promoting Campus, utilizing the Okanagan Charter Framework, which emphasizes the interconnectedness of people, places and planet in advancing health, well-being and sustainable communities (Okanagan Charter, 2015). Occupational performance, according to the PEOP model, is an important aspect of a person’s overall health and well-being. The PEOP model is a top-down approach that starts with a narrative or subjective account and can be applied to individuals, as well as groups, organizations and communities or populations (Cole & Tufano, 2020). Use of the PEOP model allowed a multi-tiered, holistic approach to the occupational well-being of the campus place, as well as its people.

### 1.4. Purpose

While evidence suggests that higher education institutions require more comprehensive support to address student well-being and academic participation, occupational therapy remains an underutilized resource within campus support systems. WILL Thrive was developed to identify areas where occupational therapy services could feasibly be integrated within existing campus support services. In alignment with the settings-based Okanagan Charter and PEOP frameworks, the program was created to support students in both living and learning contexts through a partnership with the campus wellness center and office of accessibility services.

The purpose of this program description and implementation case study was to examine steps taken to integrate occupational therapy on a college campus through a Level II occupational therapy fieldwork placement, to determine the feasibility of sustaining this program on campus and to provide insight to other higher education institutions looking to support occupational therapy fieldwork programs on their campuses. Through needs assessment activities (surveys, observations, stakeholder interviews) and process evaluation (service delivery tracking, referral patterns, resource utilization) the following research questions were examined:

1. Would occupational therapy services be utilized by students and recommended by staff, and if so, which students would most likely utilize the service?
2. How can an OT FWII student fit within the already existing services on campus?
3. What is the practicality and feasibility of creating programs that place OT FWII students on college campuses to work with students, faculty and staff?

## 2. Materials and Methods

### 2.1. Study Design

This program description and implementation case study of a new OT program (WILL Thrive) on a campus in the Northeast US examined the implementation of occupational therapy services during a 12-week Level II fieldwork placement. This study utilized an implementation evaluation approach guided by Bowen et al.’s (2009) framework to systematically document program development, implementation processes, and initial service utilization patterns. Rather than testing feasibility for broader implementation, this study aimed to describe the developmental process of creating and integrating an occupation-based wellness program within existing campus structures. Program evaluation was designed around four key feasibility domains: acceptability of OT services to students and staff, demand for OT services, implementation of OT services within existing structures, and practicality given available resources (Bowen et al., 2009). The OT program was designed with feedback from multiple stakeholders to support students on campus through an OT informed lens. Data collection involved needs assessment activities (surveys, observations, stakeholder interviews) and process evaluation (service delivery tracking, referral patterns, resource utilization). Qualitative data were descriptively coded and analyzed to support the structured integration of stakeholder perspectives within the feasibility domains of acceptability, demand, implementation, and practicality. The purpose was to determine whether and how OT services could be integrated into campus life, identify barriers to implementation, and provide recommendations for future program development.

### 2.2. Case Setting

The WILL Thrive program was developed at Russell Sage College (RSC) a small private higher education institution in the Northeast United States, during the first 12 weeks of the fall 2025 semester. The college serves undergraduate and graduate student populations with a mix of traditional (18–21) and non-traditional (25+) students, especially within the graduate programs. Total enrollment at the time of the study was under 3500. In 2019, the college integrated a campus-wide health and well-being initiative called thrive@RSC to support all campus stakeholders, building on the institution’s strengths and background in health sciences. Through this well-being initiative, the college became a member of the United States Health Promoting Campuses Network (USHPCN) and an adopter of the Okanagan Charter in October 2022, designating it as a Health Promoting Campus. The Okanagan Charter is an international charter and framework with calls to action to embed health promotion into the culture of higher education institutions while fostering health promotion action within and beyond the campus, in efforts to improve the well-being of people, their places and the planet (Okanagan Charter, 2015). Efforts to encourage cross-campus collaboration were supported by the college’s administration through the hiring of a leadership position that chairs the well-being initiative and over-sees both the campus wellness center and accessibility services, as well as recreation and fitness. This leader served as the site supervisor for the 12-week occupational therapy fieldwork II experience, providing the OT fieldwork student with access to resources, staff and students served in areas focused on living (student life, recreation, wellness center) and learning (accessibility services) in support of integrating well-being in both living and learning spaces under the college-wide thrive@RSC initiative (WILL Thrive).

### 2.3. Implementation of the WILL Thrive Program

The WILL Thrive program was completed in two phases: (1) needs assessment and (2) program development and implementation. During the first four weeks of the OT FWII experience, a needs assessment was conducted, creating a narrative account of the institution as a holistic setting in alignment with the Okanagan Charter’s whole system approach and the PEOP top-down model. The OT FWII student recorded environmental observations through interactions across campus and within both the Wellness Center and Accessibility Services Office, including formal and informal staff meetings, meetings with the site supervisor and OT faculty advisor, and observed processes within departments. Observations also included a structured environmental scan of spaces and services offered on campus that could support or hinder occupational performance. Observations were recorded and reported in an OT needs assessment template, submitted by the OT fieldwork II student in a 27-page needs assessment assignment document, which was reviewed by the OT supervisor, site supervisor and OT FW educator.

The needs assessment conducted during this initial phase of the WILL Thrive program was guided by the Okanagan Charter’s third key principle, which calls for the use of participatory approaches and engagement of student and campus stakeholder voices (Okanagan Charter, 2015). This engagement of the campus voice was created through staff, faculty and administrator stakeholder semi-structured interviews (8 total), targeted student participatory approaches of 188 first-year students across nine sessions of a 101-core curriculum course and review of 39 responses to an occupational therapy assessment (OHETI). Additionally, the OT FWII student observed 11 interactive meetings between students and accessibility services staff. These meetings are required for students to gain academic adjustments due to disability, injury or illness under the requirements.

During phase two, targeted interventions were developed and integrated (weeks five through twelve), with ongoing stakeholder interviews. Interventions and additional interviews were informed by needs assessment findings and aimed to support occupational performance and overall well-being at both the individual and settings-based levels through tiered systems of support (Hunt, 2021). Following the study’s theoretical frameworks (Okanagan Charter and PEOP model) and indicators from the needs assessment, services were organized using a multi-tiered system of support to address the identified needs of the whole student body and campus community. This tiered intervention approach is consistent with a multi-tiered systems of supports (MTSSs) framework, incorporating universal (Tier 1), targeted (Tier 2), and individualized (Tier 3) supports to address student needs across levels of intensity in alignment with a whole campus approach. The tired models were created in alignment with the Okanagan Charter’s Call to Action items.

Intensive, individualized Tier 3 interventions were provided through individual occupational therapy (OT) sessions for students who independently sought services following educational outreach or through referrals from wellness and accessibility services. These interventions were guided by Okanagan Charter Call to Action 1.4: support personal development by creating opportunities to build resilience, competence, personal capacity and life-enhancing skills.

Targeted Tier 2 interventions were identified during the needs assessment as an established service within accessibility services (Weekly Accountability Meetings) that could be enhanced through an occupational therapy lens. This demand was identified through collaborative student identification and intervention planning between the OT FWII student and accessibility services staff. These OT interventions were framed from Okanagan Charter Call to Action 1.2, “Create supportive campus environments…to enhance campus environments, identifying opportunities to support health and well-being,” (p. 7).

Data from the Occupations in Higher Education Transition Inventory (OHETI), an occupational therapy assessment utilized during the needs assessment, were reviewed to identify occupational domains warranting further programmatic exploration. The OHETI utilizes the theory of occupational adaptation to assess how well students are adapting to the daily life demands of college. The tool examines ten key areas of college life occupations through a Likert-scaled self-report, measuring three critical constructs: effectiveness, efficacy and satisfaction (K. Keptner et al., 2026b).

Assessment data from the OHETI assessment indicated that the majority (92%) of the student sample (*n* = 39) rated their current religious or spiritual participation from “just getting by” to “struggling”. These findings informed the development of Tier 2 targeted check-in emails to students registered with accessibility services, which included links to campus-based spiritual resources such as the spirituality center and chaplain. Based on this needs assessment data, the campus chaplain was identified as a relevant stakeholder and invited to participate in a stakeholder interview to further inform program development related to spirituality and well-being. Insights from this interview informed the development of Tier 1 population-level interventions, including campus-wide mindfulness-based yoga sessions aimed at promoting awareness of spirituality as a dimension of health and well-being and to connect to the larger campus culture of well-being and care as a Health Promoting Campus. These OT population level interventions were designed in alignment with the Okanagan Charter Call to Action 1.3, “Generate thriving communities and a culture of well-being” through “proactive and intentional creation of empowered, connected and resilient campus communities that foster an ethic of care, compassion, collaboration” (p. 7).

Process evaluation occurred across both phases, with data collected and analyzed during the needs assessment to identify service gaps and inform the development and implementation of occupational therapy services. Ongoing process evaluation was conducted through weekly and as-needed supervisory meetings between the OT FWII student and OT faculty advisor. These meetings included structured reflection on campus observations and stakeholder interactions using a health-promoting occupational lens, examination of contextual factors influencing implementation, and systematic review of facilitators, barriers, and intervention planning progress. The OT student completed a daily log to meet weekly objectives in tracking progress. These logs were later reviewed during process evaluation to ensure objectives were met and to further analyze the 12-week program development and implementation. Throughout all phases of the program, process evaluation data were triangulated through the three higher education professionals working with the OT FWII student: the site supervisor (campus Director of Wellness and Accessibility Services), the OT fieldwork supervisor (OTP working in Accessibility Services at a neighboring higher education institution), and the faculty supervisor (OT faculty, Assistant Fieldwork Coordinator). Open communication remained throughout the fieldwork experience and on an as-needed basis as service gaps were identified and new directions for OT services were developed. The OT faculty advisor served as a liaison to corroborate and integrate findings on program progress and effectiveness from varied viewpoints and positions, including directors of programs on campus, administrators, staff, faculty, site supervisor, OT fieldwork educator and OT student. Meetings were held with the OT faculty advisor and site supervisor on a weekly basis to review program development and implementation and to triangulate findings. Formal analysis meetings between OT faculty and OT fieldwork supervisor occurred at weeks 3, 6, 9 and 12. Finally, the OT fieldwork student created a 27-page Needs Assessment Report as a fieldwork assignment, which was reviewed for accuracy and triangulated by the OT fieldwork supervisor, OT site supervisor and OT faculty supervisor.

The OT FWII student, faculty advisor, and site supervisors were actively involved in the development, implementation, and monitoring of the WILL Thrive program, which introduces potential for confirmation bias and influences on data collection and interpretation. To mitigate this, triangulation across multiple perspectives was employed, including input from staff, faculty, and administrators not directly involved in the program, structured field notes, and regular reflective discussions among the OT FWII student, fieldwork supervisor, and faculty advisor. These steps were taken to ensure that interpretations of data were informed by multiple viewpoints and to reduce the influence of insider bias on feasibility exploration.

Table 1 provides an overview of how research methods were structured across program phases in alignment with Bowen et al.’s (2009) feasibility aspects of acceptability, demand, implementation and practicality, including corresponding data sources, methods, and outputs used to inform the different phases (needs assessment, intervention development, and process evaluation). This phased approach illustrates how multiple data sources were integrated to address the study’s research questions related to acceptability, demand, and implementation feasibility.

### 2.4. Sampling and Recruitment

Purposive sampling was used to recruit participants, including students who were perceived to benefit from OT services on campus, as indicated from the literature (first-year students and students working in accessibility services and the wellness center) and relevant stakeholders involved in student success and campus well-being. Inclusion criteria included currently enrolled students and currently employed staff on the RSC campus. Purposeful sampling is valued in qualitative research to select participants who most accurately understand a research phenomenon, providing information-rich, contextualized data (Creswell & Creswell, 2018).

Participants included first-year undergraduate students, students with identified accommodations through the Accessibility Services Office, and students referred by the wellness center. Staff participants were recruited from a variety of student support services to triangulate data, including the offices of Wellness, Accessibility Services and Student Success. Titles of stakeholder participants included mental health counselors, nurses, student success advocates, accessibility services personnel and campus chaplain. Table 1 displays participant data sources used, including 39 OHETI assessments, 8 staff interviews, five students attending at least one individual OT session, and four WAM participant students.

#### 2.4.1. Student Recruitment

During the initial needs assessment phase of this program, 188 first-year undergraduate students were introduced to the WILL Thrive model during nine sections of a first-year required core curriculum (101) course. The role of occupational therapy on a college campus was presented to students in collaboration with presentations from a variety of student support services staff, including wellness, accessibility services and recreation and fitness. The theme of this undergraduate course was titled “Thriving at RSC”, connecting students to the college-wide health and well-being initiative and integrating OT services into these offerings.

In this course, first-year students were asked to participate in a voluntary assessment tool, the Occupations in Higher Education Transitions Inventory (OHETI). Students provided electronic informed consent prior to completing the OHETI and were informed that participation was voluntary, with the option to share their de-identified, aggregated responses with the college for program development purposes. Participants received an incentive of a $10 Amazon gift card for completing the assessment. Students were also offered the opportunity to participate in a larger OHETI research study aimed at creating standardization and validation of the OHETI, which was separate and unrelated to the scope of this program description and implementation study. Students who agreed to be included in the larger study were asked to answer a few more questions and were entered into a lottery with a chance to win $100.

Students were provided two weeks to complete the OHETI and a reminder before the close. To mitigate potential response bias through the use of financial incentives, incentives for this study were modest, not contingent on responses, and participation was voluntary. Students could also choose to complete the assessment independently for personal use, without sharing data or being entered into any research study. Of the 47 students who completed the OHETI assessment, 39 of them agreed that their de-identified data could be shared with the college for reporting purposes. For the purposes of this manuscript, only de-identified, aggregate OHETI responses from these 39 students were used to inform program development; no individual-level or validation study data were analyzed here. These factors were intended to mitigate the risk of undue influence on participant responses.

Individual OT sessions were additionally advertised in a flyer displayed in and around the wellness center, at an opening day wellness tabling event, and in the accessibility services offices. The OT FWII student also educated various departments and professionals on campus about OT services, the OT scope of practice and how OT can benefit students for referral sources for student recruitment for OT services. Finally, an additional 390 students registered with accessibility services were informed about OT services through weekly targeted check-in emails. From this outreach, seven students expressed an interest in individual occupational therapy sessions, and five received individualized services. The recruitment strategy for individual OT services may have introduced selection bias, as participation relied on self-selection and referrals through existing campus systems. Multiple recruitment pathways were used to mitigate potential selection bias; however, as this study was designed as a program description exploratory feasibility case study, recruitment through existing campus systems was intentional to assess real-world integration and demand.

#### 2.4.2. Staff Recruitment

A total of eight staff members from four campus departments participated in semi-structured interviews during the needs assessment and program development phases. Data were collected across multiple disciplines to gain a broad cross-campus perspective and analyzed for descriptive themes until data saturation was reached. Participants were asked about their role in working with students and their vision of how occupational therapy can serve their students. Before being asked questions, clinicians were introduced to what occupational therapy is and how occupational therapy practitioners (OTPs) can assist college students with their success in college. Questions were aligned with Bowen et al.’s (2009) feasibility framework to address identified service gaps, monitor acceptability and demand and to inform implementation of OT services. Sample questions include the following:

- Do you feel like you are helping students in ways that land outside of your scope of practice?
- Do you see a high number of students who fall within the neurodivergent category?
- Is there a common theme to the tools that students are seeking? What is it?
- Do you feel that occupational therapy could help the students that you see?
- Would you consider referring students to occupational therapy? Why or why not?

### 2.5. Data Collection and Analysis

Data were collected throughout the needs assessment, program development and implementation phases of the study. Consistent with Bowen et al.’s framework, data sources were selected to assess accessibility and demand, inform implementation and evaluate feasibility. Data collection involved needs assessment activities (surveys, observations, stakeholder interviews) and process evaluations (service delivery tracking, referral patterns, resource utilization). Data were collected through multiple perspectives to gain a comprehensive analysis of campus needs, service gaps and feasibility of occupational therapy integration.

Table 1 outlines key methods and data collection occurring in the needs assessment phase one, which include environmental observations, semi-structured stakeholder interviews, participatory presentation in nine first-year core curriculum class sections, first-year OHETI assessments and process evaluation throughout program development and implementation. Stakeholder interviews explored perceived service gaps, student needs and the relevance of the potential role of OT services. Interview questions were informed by Bowen et al.’s (2009) domains to assess demand, acceptability and implementation considerations. Observational data were used to identify environmental facilitators and barriers to student participation and were interpreted using the PEOP model to examine interactions among person factors, environmental contexts and occupational performance demands. Findings were subsequently mapped onto the Okanagan Charter to contextualize results within a health-promoting campus framework and inform intervention development.

Process evaluation occurred through service delivery tracking, resource utilization, referral tracking and ongoing collaboration between the OT faculty advisor, site supervisor, OT fieldwork educator, OT fieldwork student and relevant staff, faculty and administrators involved in the WILL Thrive program. The OT fieldwork student recorded observations and synthesis of supervision meetings in a daily log, which tracked weekly fieldwork objectives.

Data analysis followed an interactive, descriptive approach consistent with feasibility and program evaluation research. Multiple data sources were analyzed and integrated to identify service gaps, inform intervention development, and assess feasibility within the campus context.

Daily service logs were analyzed during process evaluation to ensure objectives were met and aligned with Bowen’s feasibility measures. These logs were reviewed interactively to monitor service delivery patterns, resource utilization, and alignment with feasibility demands (acceptability, demand, implementation, and practicality). Qualitative data from stakeholder interviews and observational field notes were reviewed by researchers and coded to identify recurring themes related to service gaps and opportunities for OT intervention. Observational and environmental scan data were synthesized descriptively to identify patterns in environmental support and barriers and then mapped into feasibility domains. Quantitative aggregate data from the OHETI were analyzed descriptively to identify common areas of challenge in student occupational performance. These findings were used to prioritize intervention targets. Data were integrated across sources through triangulation and ongoing collaboration among the OT FWII student, OT faculty advisor, OT fieldwork educator and OT site supervisor, documented and analyzed within a 27-page Needs Assessment Report.

Researchers in this study received Institutional Review Board approval for secondary analysis of data collected from human participants. Participation in all program activities was voluntary, and confidentiality was maintained throughout. Students who completed the OHETI assessment provided electronic consent. Service data (session notes, referrals and utilization metrics) were reviewed only after completion of services as aggregate, de-identifiable data and no individual-level consent was obtained prior to service delivery, consistent with IRB guidance for use of operational program data in de-identified form.

## 3. Results

This program description and implementation case study employed process evaluation methods to identify campus needs and inform the development of an occupational therapy service model through a Level II fieldwork placement. Data collection was guided by Bowen et al.’s (2009) feasibility framework domains of acceptability, demand, implementation, and practicality. Qualitative data from stakeholder interviews, field notes, and observational logs were analyzed using an interactive thematic analysis approach: the OT FWII student initially coded data, which was then reviewed and triangulated with the OT faculty, site supervisor and OT fieldwork educator to ensure consistency and minimize bias. Service utilization and referral data were aggregated and descriptively summarized to support feasibility domains. Findings were synthesized to inform the development of a tiered OT service model. Results are organized around research questions one and two and corresponding feasibility domains of acceptability, demand and implementation.

### 3.1. Acceptability and Demand

*Would occupational therapy sessions be utilized by students and recommended by staff, and if so, which students would most likely utilize the service?*

Guided by Bowen et al.’s feasibility domains, research question one examined acceptability and demand for occupational therapy services on campus, including student utilization, staff referral patterns, and characteristics of students who engaged in services. The WILL Thrive program showed preliminary indicators of acceptability through utilization of OT services referred by staff in wellness and accessibility departments. Among the students who sought OT services, most presented with symptoms of ADHD (four out of five students, 80%) associated with functional challenges in academic and/or college life participation.

Weekly Accountability Meeting (WAMs) provided additional preliminary indicators of demand for OT-informed services, with the majority of participating students identified as experiencing participation-related challenges associated with diagnosed autism spectrum disorder (three of four, 75%) and anxiety (three of four, 75%). Both undergraduate and graduate students participated in OT services.

#### 3.1.1. Individual OT Sessions

Individual occupational therapy sessions were structured to support students’ engagement in meaningful academic and daily life occupations within the college context. Initial 60 min sessions included development of an occupational profile specific to the college student, including exploration of students’ roles, routines and habits and environmental demands related to their academic and personal life participation. Collaborative goal setting targeted student-identified occupational barriers. Follow-up 45 min OT sessions included student self-reflection and reported updates on goal-related activities, with interventions designed to support continued engagement. Field note data documented the use of occupation-based interventions to structure daily academic and life routines, support emotional regulation during academic and social occupations, and promote attentional regulation and task engagement.

Seven students expressed interest in scheduling an individual OT session. Six students scheduled an initial OT session, with one no-show. Of the five students who attended an initial OT session, four students scheduled a follow-up session. Three students attended the follow-up session, with one no-show. Participation and attrition data are summarized in Table 2.

Of the five students who participated in individualized occupational therapy intervention, three (60%) were undergraduate students (first year, sophomore and junior) and two (40%) were graduate students. Four out of five students reported attentional challenges consistent with ADHD symptoms (80%), including one student without a formal diagnosis who sought services for these concerns. Two of the four students reported a late diagnosis of ADHD (after secondary education) (50%). Participant characteristics are presented in Table 3.

#### 3.1.2. Weekly Accountability Meetings (WAMs)

Weekly Accountability Meetings (WAMs) were facilitated by the OT FWII student as part of an established accessibility services support model. While accessibility services previously offered general check-ins for students, OT WAMs differed by incorporating a structured occupational therapy lens, emphasizing occupation-based goal setting, strategy development, and contextual support for both academic and daily life participation in alignment with the PEOP model.

Based on observational data, stakeholder input, and collaborative process evaluation, the OT FWII student conducted WAMs with four students registered with accessibility services. Field notes documented collaborative goal setting focusing on social participation, skill development, and strategies to support occupational engagement in academic and college life.

Three out of the four students (75%) attended OT WAMS consistently, each completing a minimum of three follow-up sessions. Compared with the five students who received individual OT sessions, WAM participants attended more frequently and consistently. These patterns describe attendance trends within this small sample size, suggesting that structured, less intensive Tier 2 interventions embedded within the existing campus support system may support repeated engagement. While these attendance patterns indicate potential demand, the small number of participants warrants interpretation as preliminary signals of feasibility rather than definitive evidence of utilization trends.

Students participating in WAMs reported a range of co-occurring neurodevelopmental and mental health concerns, including autism spectrum disorder (ASD) and anxiety (*n* = 2, 50%) and attention-deficit/hyperactivity disorder (ADHD), anxiety, insomnia and dyslexia (*n* = 1, 25%). Participant demographics are presented descriptively in Table 4.

### 3.2. Implementation and Service Delivery Model

*How can an OT FWII student fit within the already existing services on campus?*

Research question two examined implementation, including how an OT FWII student was integrated within existing campus services and how occupational therapy services were feasibly delivered within the current system. Occupational therapy services were delivered using a multi-tiered model of support, informed by the needs assessment findings and ongoing process evaluation, and guided by the Okanagan Charter Framework Calls to Action. The WILL Thrive program included OT interventions implemented across three tiers of support, including individualized (Tier 3: individual OT sessions), targeted (Tier 2: WAMs, weekly check-in email) and Universal (Tier 1: population-level groups, resource list) services. Findings related to implementation addressed how an OT FWII student could be integrated within existing campus services. Table 5 outlines the types and frequency of OT services delivered across tiers throughout the program implementation period and their relation to the Okanagan Charter Framework Calls to Action. This table demonstrates how a multi-tiered approach allowed the OT FWII student to deliver individualized, targeted, and population-level interventions, supporting both the feasibility of service delivery and alignment with the Okanagan Charter framework.

## 4. Discussion

This program description and implementation case study examined the feasibility of WILL Thrive, an occupational therapy program delivered on a college campus through a Level II fieldwork placement. Consistent with Bowen et al.’s (2009) feasibility framework, findings related to research question three provide early, exploratory indicators of practicality and implementation feasibility for a fieldwork-based occupational therapy model within a higher education setting. Early indicators of acceptability of services were observed through stakeholder engagement and referral behaviors, while signals of demand were reflected in student utilization of services, although participation numbers were small. Services were accessed by students reporting functional challenges related to academic participation, including students with reported neurodivergent profiles (ADHD, ASD) and mental health concerns such as anxiety; however, the sample size and variability limit conclusions regarding a consistent service-user profile. Although the program was initially conceptualized within a broad campus wellness framework, referrals and service utilization in this case were most frequently observed among students reporting neurodevelopmental and mental-health related challenges; however, these patterns should be interpreted as preliminary and context-specific rather than indicative of a defined target population. These findings should be interpreted cautiously as early indicators of feasibility, with recommendations for future research to establish robust evidence of established demand. Program evaluation provides preliminary indicators that an occupational therapy fieldwork student can contribute meaningful services to target gaps in behavioral and academic support on campus, while supervision requirements emerged as a key implementation barrier impacting sustainability.

The discussion is organized around key feasibility domains, including acceptability and demand, integration within existing campus services, and implementation feasibility, situated within a Health Promoting Campus framework.

### 4.1. Alignment with a Health Promoting Campus Framework

An important asset in the implementation of WILL Thrive was its alignment with an established Health Promoting Campus framework and systems-based approach to campus-wide well-being. Occupational therapy’s focus on both living and learning settings and occupations aligns with systems-level approaches to health promotion, positioning OTPs as potential connectors across current campus support systems. This role is consistent with the Okanagan Charter’s (2015) second principle for action, which emphasizes the development of comprehensive and campus-wide approaches to health promotion, as well as the fourth principle, which calls for trans-disciplinary collaboration. By working collaboratively across departments, occupational therapists may serve as a bridge profession supporting integrated, sustainable campus well-being initiatives that extend beyond individual service delivery to influence interconnected institutional systems.

Despite these assets, challenges related to a limited understanding of OT services emerged, echoing prior research indicating that the implementation of occupational therapy in higher education requires substantial education due to confusion regarding OT’s role and scope (Eichler & Keptner, 2023). Consistent with the literature, findings suggest that education regarding OT should be directed toward students, faculty, and institutional staff to support appropriate referrals and coordinated service delivery (Eichler & Keptner, 2023).

### 4.2. Acceptability and Demand for Campus-Based OT Services

The WILL Thrive program demonstrated preliminary indicators of acceptability, reflected in utilization patterns and staff referral behavior, primarily by staff in wellness and accessibility departments. Demand was observed among students experiencing functional challenges related to executive functioning, social participation, emotional regulation, self-advocacy and role participation within academic contexts, consistent with documented barriers to academic success among students with ADHD, ASD and anxiety (Anderson et al., 2017; Henning et al., 2022; Palomares Gómez et al., 2023; Dwyer et al., 2023). Given the small, heterogeneous sample, these trends should be considered preliminary and suggest promising areas for future study rather than definitive patterns of service use.

Notably, students sought OT services to support participation in daily activities associated with college life and academics rather than to clarify or obtain a diagnosis, reinforcing the relevance of occupation-focused intervention for students who may lack, delay or avoid formal disability disclosure. This finding suggests a potential role of OT in addressing functional needs among students without documented diagnoses, particularly given evidence of high rates of undiagnosed or late diagnosed conditions, especially ADHD (Barnard-Brak, 2025), as well as documented reluctance to disclose diagnoses to faculty (Mamboleo et al., 2019). By embedding outreach within both “living” support services (Wellness Center) and “learning” support services (Accessibility Services), the WILL Thrive program may offer a complementary approach to supporting students who may not seek accommodations or feel comfortable disclosing disabilities. These preliminary indicators should be interpreted cautiously due to the lack of comparative analysis within existing services.

Although the program was initially conceptualized within a broad campus wellness framework, referrals and service utilization in this case were most frequently observed among students reporting neurodevelopmental and mental health-related challenges; however, these patterns should be interpreted as preliminary and context-specific rather than indicative of a defined target population. Within this case context, the concentration of referrals among students reporting neurodevelopmental and mental health-related challenges may suggest that existing campus wellness approaches do not fully address the functional and participation-based needs of all student groups, particularly those navigating neurodivergence; however, this interpretation should be considered exploratory and warrants further investigation.

The recruitment strategy for individual OT services may have introduced selection bias, as participation relied on self-selection and referrals through existing campus systems. As a result, students who engaged in services may have been more connected to campus resources or more receptive to these supports, limiting generalizability to the broader student population.

### 4.3. Integration Within Existing Campus Services

#### 4.3.1. Occupational Therapy’s Unique Contribution in Higher Education

While structured support was provided on campus, study findings suggest that occupational therapy may complement existing services by targeting functional, contextual and participation-based needs not fully met through counseling or academic accommodation alone. This was observed by students who utilized both the WAMs and OT sessions, consistent with the emerging literature describing OT’s expanding role in higher education settings (Eichler & Keptner, 2023; Hojati Abed et al., 2021; Hunt et al., 2025). These findings should be regarded as preliminary indicators due to the small sample size.

The individualized person–environment–occupation approach utilized within WILL Thrive interventions reflects occupational therapy’s capacity to support student self-determination, routine development and occupational balance, which are outcomes increasingly recognized as central to student well-being and success. The WILL Thrive program saw greater continuity of participation within the already established structure of WAMS, suggesting that integration into familiar service models may support sustained engagement. The regularity of weekly meetings provided structure and time to build upon functional skills supporting challenges commonly experienced by students with neurodevelopmental differences, including time management, sleep hygiene, self-advocacy and social participation (Anderson et al., 2017; Dwyer et al., 2023). These findings align with evidence that academic success among postsecondary students with disabilities is supported by integrated health-promotion approaches that connect students to peers, social activities, and faculty and staff support (Römhild & Hollederer, 2024).

#### 4.3.2. OT as a Complementary Campus Service

Referral patterns provide preliminary indicators of occupational therapy’s potential as a complementary role within campus service systems, with wellness and accessibility professionals identifying OT as an appropriate referral service when students’ needs extend beyond the traditional scope of counseling or academic accommodations. This supports the existing literature positioning occupational therapy as a collaborative campus partner rather than a duplicative service (Eichler & Keptner, 2023; Ho, 2022; K. Keptner et al., 2026a; Lewis, 2025).

### 4.4. Feasibility, Assets, and Implementation Challenges

#### Fieldwork as a Feasible but Constrained Delivery Model

Preliminary findings suggest that a Level II occupational therapy fieldwork student may be able to implement OT services within a campus setting, with services utilized and perceived as valuable by students and staff; however, participation was limited, and findings remain exploratory. Challenges related to supervision requirements emerged, consistent with reported constraints in fieldwork education and the limited ability of OTPs to supervise in emerging practice settings such as a college campus (Eichler & Keptner, 2023; Evenson et al., 2015; Varland et al., 2017). Embedding an occupational therapy fieldwork II student within campus services aligns with emerging recommendations for health promotion, early intervention and prevention-oriented, occupation-based approaches that increase accessibility in higher education (Hunt et al., 2025 Nagata et al., 2022) and may contribute to improved degree completion (Henning et al., 2022). While fieldwork-based models may serve as an entry point for introducing OT services on a college campus, supervision requirements present sustainability challenges, suggesting that institutional investment in occupational therapy roles on campus may be necessary for long-term implementation.

### 4.5. Broader Implications and Research Limitations

#### 4.5.1. Practical Implications for Campus OT Programs

Future implementation of the WILL Thrive program or similar models placing an OT FWII student within a college campus setting should emphasize campus specific needs assessment, stakeholder education, interdepartmental collaboration, and occupation-based, contextually grounded interventions. Fieldwork-based delivery models may be particularly valuable for institutions unable to immediately hire full-time occupational therapy practitioners, provided supervision challenges are addressed through institutional planning and investment.

Future implementation of campus-based OT services through programs like WILL Thrive should include four core elements:

- **Evaluate** campus specific needs and existing supports to determine appropriate fit for OT services;
- **Educate** stakeholders on the value and role of OT on college campuses to support referral pathways and reduce service delivery gaps;
- **Collaborate** across departments to coordinate services with comprehensive campus wide support through both living and learning environments;
- **Connect** with person(s) and environment, grounding work in OT and health promotion frameworks in creating whole-person, environment-responsive and population-level interventions.

**Figure 1:**
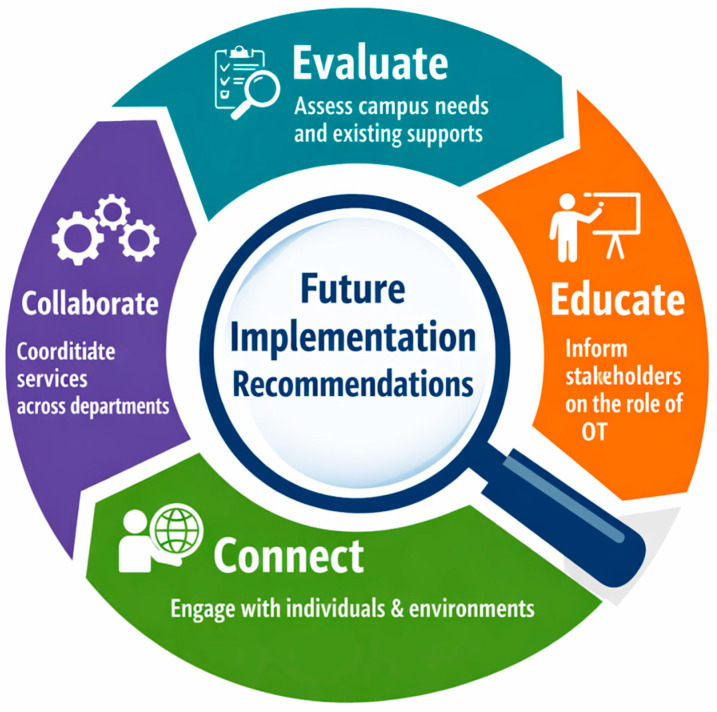
Will Thrive program recommendations roadmap.

Figure 1 provides a visual representation of these four core elements of the WILL Thrive program design and process evaluation recommendations for practice.

#### 4.5.2. Directions for Future Research

Consistent with Bowen et al.’s (2009) feasibility framework, this study addresses the question of “Can it work?” within a single small institution in the Northeastern United States. As a single-site, exploratory case study with a 12-week implementation timeline, findings are intended to document process, program development and preliminary feasibility rather than to establish generalizable outcomes. Findings should be considered as process documentation and lessons learned to inform future program development efforts in similar contexts.

Future research should examine “Does it work” as outlined by Bowen et al.’s (2009) framework through pre-post outcome measures assessing occupational performance, participation, well-being and academic engagement, as well as “Will it work” across diverse campus contexts. While limited by the small sample size and contextual specificity, these findings remain informative as early indicators for feasibility for institutions exploring occupational therapy integration within higher education. Further research across diverse institutional contexts is warranted to examine the generalizability and scalability of occupational therapy fieldwork programs within higher education.

#### 4.5.3. Study Limitations

Study limitations include the absence of formal outcome measures, which preclude conclusions regarding student-level changes in occupational performance, participation, or well-being. Recruitment relied heavily on self-selection and referral pathways aligned with existing services, which may introduce some selection bias and limit the representativeness of the participants. Analytic procedures, while systematic and triangulated, were descriptive and qualitative and do not allow for strong causal inferences. The close involvement of the OT FWII student, faculty advisor and supervisors may have introduced confirmation bias, despite efforts to mitigate this through triangulation and reflective discussions. Furthermore, it is difficult to separate the feasibility of providing occupational therapy on a college campus in general from the feasibility of this specific supervision and fieldwork delivery model. As such, findings should be interpreted as early signals of feasibility rather than robust evidence of effectiveness or generalizability.

## 5. Conclusions

The WILL Thrive program provides preliminary insights into the potential role of occupational therapy as a complementary support service within higher education settings through a Level II fieldwork placement. This program description and implementation case study offers early signals of acceptability and potential demand, based on patterns of stakeholder referral and student utilization within a single site, small-sample context. Students who engaged in services included those reporting attention-deficit/hyperactivity disorder, autism spectrum disorder, and anxiety; however, the sample size limits the generalizable service-user profile. These preliminary observations suggest that students experiencing neurodevelopmental and mental health-related participation challenges may represent a promising area for future investigation.

By supporting occupational balance across both living and learning contexts, campus-based occupational therapy may offer a complementary approach to addressing participation challenges; however, future research should explore occupational therapy services along with outcomes to determine effectiveness across diverse higher education contexts.

## Figures and Tables

**Table 1:** Application of Bowen et al.’s feasibility framework to the WILL Thrive program.

Feasibility Domain (Bowen et al., 2009)	Program Phase	Purpose	Key Methods	Primary Outputs
**Acceptability and Demand**	Phase 1: Needs Assessment (Weeks 1–4)	To examine access to campus environments and demand for OT services	Environmental observations across campus settings Structured environmental scan of spaces and services Staff, faculty, and administrator stakeholder interviews (7) Student participatory activities in first-year course Review of 39 OT OHETI assessment data Observation of Accessibility Services interactive meetings	Identification of service gaps; participation barriers; stakeholder interest in OT services
**Implementation**	Phase 2: Program Development and Integration (Weeks 5–12)	To develop and integrate OT-informed interventions responsive to identified needs	Development of targeted and population-level interventions; (4 WAM Tier 2 and 5 individual Tier 3 OT sessions) Continued stakeholder interviews (1) Collaboration with campus partners (e.g., campus chaplain, recreation and fitness departments)	Tiered OT interventions; integration into campus systems; interprofessional collaboration
**Feasibility (Process Evaluation)**	Across Phases 1–2	To assess practicality, fit, and refinement of OT services	Weekly and as-needed check-ins between OT FWII student and OT faculty advisor Triangulation of perspectives from site supervisor, OT fieldwork educator, and faculty supervisor Ongoing review of facilitators, barriers, and contextual constraints Field note tracking of service delivery patterns, referral patterns, and resource utilization Refinement of services guided by needs assessment data and ongoing identification of service gaps	Identification of implementation facilitators and barriers; institutional buy-in; feasibility of Level II fieldwork model

**Table 2:** Student interest and participation in individual OT sessions.

Interested	Scheduled Initial	Attended Appt	Scheduled F/U	Attended F/U
7 (100%)	6 (86%)	5 (71%)	4 (57%)	3 (43%)

**Table 3:** Demographics of individual OT participants.

Participant	Academic Status	Reported Diagnosis/Concern	Number of Visits
Student 1	Sophomore	Self-reported ADHD (no formal diagnosis)	2
Student 2	First Year	ADHD and Insomnia	1
Student 3	Junior	Anxiety	2
Student 4	Graduate student	Late-diagnosed ADHD and PTSD	2
Student 5	Graduate student	Late-diagnosed ADHD and OCD	1

**Table 4:** Demographics of WAM participants by class and Dx.

Participant	Academic Status	Reported Diagnosis/Concern	Number of Visits
Student 1	Junior	ASD, anxiety	6
Student 2	Sophomore	ASD, anxiety	6
Student 3	Freshman	ADHD, anxiety, insomnia, dyslexia	4
Student 4	Freshman	ASD	1

**Table 5:** Description of WILL Thrive OT tiered interventions.

Intervention Services	Tiered Level of Support	Description and Tie to Okanagan Charter Framework
Individual OT Sessions	Tier 3: Individual support	Eight individual OT sessions emphasizing occupational profile development, occupation-based and contextualized interventions incorporating education, environmental modifications, and strategy development to support participation in coursework, routines, and campus life *(Okanagan Charter Call to Action 1.4: Personal development support)*
WAMs	Tier 2: Targeted support	Weekly meetings for students registered with accessibility services; integrated occupational approaches to support academic participation *(Okanagan Charter Call to Action 1.2, Create supportive campus environments)*
Weekly Email	Tier 2: Targeted support	Weekly email communication to students registered with accessibility services, guided by needs assessment data, including targeted topics of school/life balance, sleep, stress management, study tools, spirituality, emotional and self-regulation *(Okanagan Charter Call to Action 1.2, Create supportive campus environments)*
Mindfulness Campus Groups	Tier 1: Universal population level support	Six weekly campus-wide mindfulness yoga sessions and one wellness day supported activity *(Okanagan Charter Call to Action 1.3 Create thriving communities and a culture of well-being)*
List of Resources	Tier 1: Universal population resource	Curated list of resources to support occupational performance of college students, specific to identified campus needs and supports *(Okanagan Charter Call to Action 1.3 Create thriving communities and a culture of well-being)*

## Data Availability

The datasets generated and analyzed during the current study are not publicly available due to institutional ethical restrictions and the potential risk of participant identification within case-based occupational therapy service notes. De-identified data were securely stored on an access-restricted Google Drive and on password-protected computers accessible only to the principal investigator and authorized student investigators. Limited data may be available from the corresponding author upon reasonable request and with institutional approval.

## Abbreviations

The following abbreviations are used in this manuscript:

OT	Occupational Therapy
OTP	Occupational Therapy Practitioner
FWII	Level Two Fieldwork
WILL Thrive	Well-Being in Living and Learning to Thrive
